# Pharmacological Management of ADHD in Older Adults: Focus on Methylphenidate

**DOI:** 10.1192/j.eurpsy.2026.11568

**Published:** 2026-07-31

**Authors:** A. Rami

**Affiliations:** Razi Hospital, Manouba, Tunisia

## Abstract

**Introduction:**

Attention-Deficit/Hyperactivity Disorder (ADHD) is increasingly recognized as a lifelong neurodevelopmental condition, persisting into adulthood and even late life. With improved diagnostic awareness, a growing number of older adults (≥60 years) are being identified as having ADHD. However, evidence regarding pharmacological treatment—particularly with methylphenidate—is limited in this age group, raising questions about efficacy, safety, and clinical appropriateness.

**Objectives:**

This literature review aims to summarize current knowledge on the use, effectiveness, and tolerability of methylphenidate in older adults diagnosed with ADHD, and to identify existing gaps in clinical research.

**Methods:**

A narrative review of studies published between 2000 and 2025 was conducted through PubMed, ScienceDirect, and PsycINFO databases. Included articles examined clinical trials, case reports, and observational studies evaluating methylphenidate or other psychostimulants in adults aged 55 years and older with confirmed ADHD diagnosis.

**Results:**

Available evidence, though scarce and heterogeneous, suggests that methylphenidate can improve attention, executive functioning, and daily functioning in selected older adults with ADHD. Response rates appear comparable to those of younger adults; however, dose requirements may be lower, and side-effect sensitivity is higher, particularly for cardiovascular and sleep-related adverse events. Studies emphasize the need for individualized dosing, gradual titration, and comprehensive cardiovascular screening prior to initiation. Long-term data remain lacking, and most trials exclude elderly patients with comorbidities, limiting generalizability.

**Conclusions:**

Methylphenidate represents a potentially effective but cautiously prescribed option for managing ADHD symptoms in older adults. Given age-related pharmacokinetic changes and increased medical comorbidities, treatment must rely on careful patient selection, close monitoring, and integration with non-pharmacological strategies. Future research should prioritize controlled trials and geriatric-specific safety data to guide evidence-based prescribing in this under-studied population.

**Disclosure of Interest:**

None Declared

